# *CXCL12* promoter methylation and PD-L1 expression as prognostic biomarkers in prostate cancer patients

**DOI:** 10.18632/oncotarget.10786

**Published:** 2016-07-22

**Authors:** Diane Goltz, Emily Eva Holmes, Heidrun Gevensleben, Verena Sailer, Jörn Dietrich, Maria Jung, Magda Röhler, Sebastian Meller, Jörg Ellinger, Glen Kristiansen, Dimo Dietrich

**Affiliations:** ^1^ Institute of Pathology, University Hospital Bonn, Bonn, Germany; ^2^ Weill Cornell Medicine of Cornell University, Department of Pathology and Laboratory Medicine, New York, NY, USA; ^3^ Weill Cornell Medicine of Cornell University, Englander Institute for Precision Medicine, New York, NY, USA; ^4^ Department of Urology, University Hospital Bonn, Bonn, Germany; ^5^ Department of Otolaryngology, Head and Neck Surgery, University Hospital Bonn, Bonn, Germany

**Keywords:** CXCL12, DNA methylation, prostate cancer, biomarker

## Abstract

**Background:**

The CXCR4/CXCL12 axis plays a central role in systemic metastasis of prostate carcinoma (PCa), thereby representing a promising target for future therapies. Recent data suggest that the CXCR4/CXCL12 axis is functionally linked to the PD-1/PD-L1 immune checkpoint. We evaluated the prognostic value of aberrant *CXCL12* DNA methylation with respect to PD-L1 expression in primary PCa.

**Results:**

*CXCL12* methylation showed a consistent significant correlation with Gleason grading groups in both cohorts (*p* < 0.001 for training and *p* = 0.034 for testing cohort). Short BCR-free survival was significantly associated with aberrant CXCL12 methylation in both cohorts and served as an independent prognostic factor in the testing cohort (hazard ratio = 1.92 [95%CI: 1.12–3.27], *p* = 0.049). Concomitant aberrant *CXCL12* methylation and high PD-L1 expression was significantly associated with shorter BCR-free survival (*p* = 0.005). In comparative analysis, the *CXCL12* methylation assay was able to provide approximately equivalent results in biopsy and prostatectomy specimens.

**Materials and Methods:**

*CXCL12* methylation was determined by means of a methylation specific quantitative PCR analysis in a radical prostatectomy patient cohort (*n* = 247, training cohort). Data published by The Cancer Genome Atlas served as a testing cohort (*n* = 498). *CXCL12* methylation results were correlated to clinicopathological parameters including biochemical recurrence (BCR)-free survival.

**Conclusions:**

*CXCL12* methylation is a powerful prognostic biomarker for BCR in PCa patients after radical prostatectomy. Further studies need to ascertain if *CXCL12* methylation may aid in planning active surveillance strategies.

## INTRODUCTION

Prostate cancer (PCa) is a highly prevalent disease that remains clinically silent in the majority of cases and is mostly curable when localized. Advanced stages of PCa initially respond well to anti-androgen therapy but will usually progress to castration resistance with poor prognosis. Obviously, new prognostic tools are needed to allow for an early recognition of aggressive versus indolent forms of PCa. Furthermore, the development of novel targeted therapies for advanced PCa, e.g. immunotherapies, necessitates the development of future predictive biomarkers as companion diagnostics.

The α-chemokine receptor C-X-C chemokine receptor type 4 (CXCR4) and its endogenous ligand CXCL12, also called stromal-derived-factor 1 (SDF1), are attractive therapeutic targets as they are widely expressed in numerous epithelial, mesenchymal, and hematopoietic tumors [1–8]. Recent data suggest a chief position of the CXCR4/CXCL12 axis initializing androgen dependent proliferation, tumor cell motility, and metastatic growth in PCa [9].

Originally, CXCL12 was found to control hematopoietic cell trafficking and guide the homing of stem cells within the bone marrow [10, 11]. Thereby, cells with CXCR4 expression migrate towards compartments with high CXCL12 production. In the bone marrow, CXCL12 signals lead to cell chemotaxis until they are terminated through CXCR4 receptor internalization [12]. In the long-run, steady CXCL12 binding causes CXCR4 desensitization resulting in a resting, non-deploying state of hematopoietic stem cells and tumor cells, likewise [13]. This implies that a microenvironment with a constant CXCL12 production induces a down-regulation of CXCR4 and thereby antagonizes the process of metastasis [10]. Gene methylation adds to the major regulatory alterations interfering with CXCL12 homeostasis in tumor epithelium, leading to a silenced CXCL12 signal in various human malignant tumors [14–17].

Moreover, the CXCR4/CXCL12 axis seems to be tightly linked to the immune checkpoint regulator programmed death 1 (PD-1) and programmed death-ligand 1 (PD-L1) that co-operate to suppress anti-cancer immunity [18]. In the clinical setting, anti-PD-1 and anti-PD-L1 antibodies have shown promising outcomes in cancer patients. However, a subset of patients fails to respond to checkpoint inhibition [19]. Feig *et al.* recently managed to overcome anti-PD-L1 drug resistance in a murine pancreatic adenocarcinoma model by blocking CXCR4. These findings motivated us to investigate the CXCR4/CXCL12 axis within the cross-relation to PD-L1 expression in PCa

## RESULTS

### Analytical assay performance

**Figure 1:**
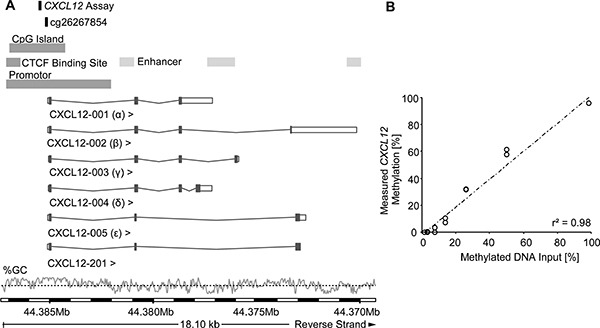
Genomic location and analytical performance verification of the quantitative *CXCL12* methylation-specific real-time PCR and the bead cg26267854 from the Illumina Infinium HumanMethylation450 BeadChip Location of the gene is on the reverse strand of chromosome 10, the information for this figure was taken from Ensembl Homo sapiens version 82.38 (GRCh38.p3). Six splice variants of *CXCL12* have been identified in humans, with CXCL12-α and CXCL12-β as the main isoforms. The GC content [%] is shown with the black dotted line indicating 50% GC content (**A**). For analytical performance verification of the qPCR assay a dilution series of bisulfite-converted artificially methylated and unmethylated sperm DNA was analysed (mixtures: 0, 0.78, 1.56, 3.125, 6.25, 12.5, 25, 50, 100 % methylated DNA). Each DNA mixture was analysed in duplicate (**B**).

Methylation levels of *CXCL12* were determined by quantitative methylation-specific real-time PCR detecting methylated and unmethylated *CXCL12* promoter sequences. The assay was designed within a CpG island upstream of the *CXCL12* gene (Figure 1A). The analytical performance of the assay was tested using mixtures of bisulfite converted artificially methylated and unmethylated DNA. Figure 1B shows that the assay allowed for the accurate quantification of *CXCL12* methylation within the whole spectrum of 0 – 100% methylation (*r*^2^ = 0.98).

### Frequency of *CXCL12* gene methylation in human prostate tissue samples

**Figure 2:**
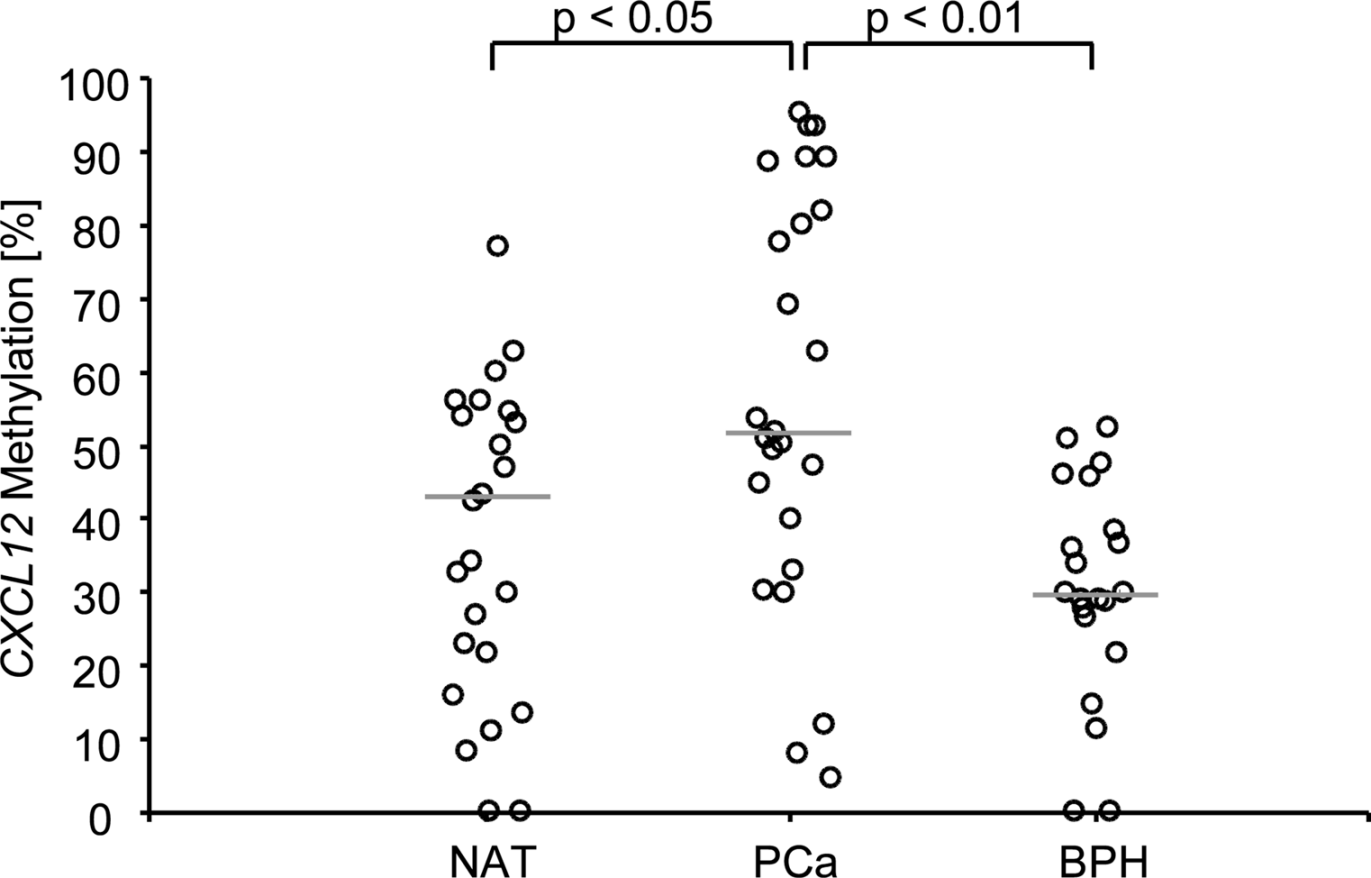
*CXCL12* promoter methylation in tissue samples from PCa patients Normal adjacent tissue samples and benign hyperplastic samples displayed a lower *CXCL12* promoter methylation than prostate cancer samples. The median methylation is indicated by the grey line. The two cut-offs used for the survival analysis are indicted by the dotted lines. In Bonferroni's multiple comparison test, PCa showed significantly higher *CXCL12* methylation percentages than normal adjacent and benign hyperplastic tissue.

In a test case control study, we evaluated a total of 66 human prostate tissue samples from 25 radical prostatectomy specimens regarding their *CXCL12* methylation (m*CXCL12*) status in normal prostate parenchyma, hyperplastic adenomyomatous tissue, and carcinomatous tissue. These 25 PCa specimens were representative of a wide spectrum of the disease and comprised the following Gleason grading groups (GG): GG I 28%, GG II-III 28%, GG IV 24, and GG V 20%. Among non-carcinomatous samples, we analyzed 22 normal tissue samples remote from the tumor front and 19 benign hyperplastic cases. Compared to PCa samples, *CXCL12* methylation was altogether significantly lower in benign and normal specimens compared to PCa samples (*p* < 0.05 and *p* < 0.01, Figure 2). Mean m*CXCL12* values of normal and PCa tissue were narrowly confined to 39.5% [31.1%-48.1%] and 56% [45.4%-68.5%], respectively. Dispersion around the mean of m*CXCL12* in PCa samples, however, was significantly more prominent (Χ^2^ = 14.1*, p* < 0.001, Bartlett's test for equal variances). In detail, one group of patients showed values offsetting over approximately 70% methylation and another group was observed at a distance below approximately 30% methylation (Figure 2).

Differences in the spectrum of m*CXCL12* prompted us to investigate the clinical performance of m*CXCL12* in two independent cohorts of patients that had undergone radical prostatectomy.

### Methylation and expression of *CXCL12* in the training cohort

Full clinicopathological characteristics of the training cohort are given in Table 1. Among prognostic clinicopathological variables, a significant association was found between high m*CXCL12* and high Gleason grading groups (*p* < 0.001) as well as nodal positive (pN1) patients (*p* = 0.038; Table 1).

**Table 1:** Clinico-pathological data and their association with *CXCL12* methylation in the training (*n* = 247) and the testing (*n* = 498) cohort comprised of prostate cancer patients who underwent radical prostatectomy

	Training Cohort						Testing Cohort					
	All Patients	Mean *mCXCL12* [%]	*p*-value	m*CXCL12*_low_	m*CXCL12*_medium_	m*CXCL12*_high_	All Patients	Mean m*CXCL12* [%]	*p*-value	m*CXCL12*_low_	m*CXCL12*_medium_	m*CXCL12*_high_
Patient number [*n*]	247 (100%)	55.8		45 (18.2%)	137 (55.5%)	65 (26.3%)	498 (100%)	7.8		156 (31.3%)	301 (60.4%)	41 (8.2%)
Patients with follow-up	216 (87.4%)						430 (86.3%)					
Mean follow-up [months]	61.3						21.9					
Median follow-up [months]	62						16.2					
Range follow-up [months]	0–145						0.6–115					
**Biochemical recurrence (BCR)**												
Yes	44	61.08					55	7.2				
Censored	172	55.04	*p* = 0.057*				362	10.6	*p* = 0.97*			
**Age**												
Mean [years]	64.13						61					
Median [years]	65						61					
≤ Median [*n*]	130 (52.6%)	56		26 (20%)	69 (53.1%)	35 (26.9%)	251 (50.4%)	6.7		91 (36.2%)	144 (57.4%)	16 (6.4%)
> Median [*n*]	117 (47.4%)	55.5	*p* = 0.223*	19 (16.2%)	68 (58.1%)	30 (25.6%)	247 (49.6%)	9	*p* = 0.004*	65 (26.3%)	157 (63.6%)	25 (10.1%)
**pT category**												
pT2	168 (68%)	54		29 (17.3%)	101 (60.1%)	38 (22.6%)	188 (37.8%)	8		61 (32.4%)	110 (58.5%)	17 (9%)
pT3 and pT4	79 (32%)	59.6	*p* = 0.041*	16 (20.3%)	36 (45.6%)	27 (34.2%)	303 (60.8%)	7.7	*p* = 0.64*	94 (31%)	186 (61.4%)	23 (7.6%)
**pT category and surgical margin**												
pT2/3a and R0	120 (48.6%)	54.7		22 (18.3%)	69 (57.5%)	29 (24.2%)	265 (52.2%)	6.3		91 (34.3%)	159 (60%)	15 (5.7%)
pT3b/c and pT4 or R1	122 (49.4%)	57	*p* = 0.31*	22 (18%)	65 (53.3%)	35 (28.7%)	203 (40.8%)	9.5	*p* = 0.005*	58 (28.6%)	123 (60.6%)	22 (10.8%)
							30 (6%)					
**Surgical margin**												
R1	86 (34.8)	56.9		12 (14%)	50 (58.1%)	24 (27.9%)	151 (30.3%)	6.4		36 (23.8%)	96 (63.6%)	19 (12.6%)
R0	156 (63.2%)	55.3	*p* = 0.40*	32 (20.5%)	84 (53.8%)	40 (25.6%)	316 (63.5%)	10.5	*p* < 0.001*	113 (35.8%)	185 (58.5%)	18 (5.7%)
unknown	5 (2%)						30 (6%)					
**Preoperative PSA**												
Range [ng/ml]	0.41–395						0.7–107					
Mean [ng/ml]	12.12						11					
Median [ng/ml]	7.17						7.5					
<4 [*n*]	23 (9.3%)	50.2		3 (13%)	16 (69.6%)	4 (17.4%)	53 (10.6%)	7.1		14 (23.4%)	37 (69.8%)	2 (3.8%)
4–10 [*n*]	150 (60.7%)	54.8		27 (18%)	88 (58.7%)	35 (23.3%)	288 (57.8%)	9.9		96 (33.3%)	164 (56.9%)	28 (9.7%)
>10	74 (30%)	59.6	*p* = 0.13†	15 (20.3%)	33 (44.6%)	26 (35.1%)	154 (30.9%)	8.3	*p* = 0.84†	154 (29.2%)	99 (64.3%)	10 (6.5%)
							3 (0.6%)					
**Gleason grading group**												
1 (< 7)	133 (53.8%)	52		26 (19.5%)	79 (69.4%)	28 (21.1%)	45 (5.7%)	5.7		13 (28.6%)	29 (64.4%)	3 (6.7%)
2 (3 + 4)	46 (18.6%)	54.3		11 (23.9%)	23 (50%)	12 (26.1%)	147 (29.5%)	5.8		60 (40.8%)	79 (53.7%)	8 (5.4%)
3 (4 + 3)	18 (56.7%)	56.7		2 (11.1%)	12 (66.7%)	4 (22.2%)	101 (20.3%)	7		28 (27.7%)	69 (68.3%)	4 (4%)
4 (8)	30 (67.6%)	67.6		4 (13.3%)	12 (40%)	14 (46.7%)	64 (12.9%)	8.6		12 (18.8%)	46 (71.9%)	6 (9.4%)
5 (> 8)	14 (5.7%)	64	*p* < 0.001†	2 (14.3%)	9 (64.3%)	3 (21.4%)	141 (28.3%)	10.9	*p* = 0.034†	43 (30.5%)	78 (55.3%)	20 (14.2%)
Unknown	6 (2.4%)						0					
**Nodal status**												
pN0	230 (93.1%)	54.9		43 (18.7%)	130 (56.5%)	57 (24.8%)	346 (69.5%)	7.4		110 (31.8%)	211 (61%)	25 (7.2%)
pN1	14 (5.7%)	68.4	*p* = 0.038*	2 (14.3%)	5 (35.7%)	7 (50%)	79 (15.9%)	8.6	*p* = 0.85*	27 (24.2%)	44 (55.7%)	8 (10.1%)
Unknown	3 (1.2%)						73 (14.6%)					

* Wilcoxon-Mann-Whitney test

† Kruskal-Wallis test.

In the training cohort, m*CXCL12* revealed a symmetric distribution covering a broad spectrum of values (0.04% – 98.40%) with a major peak seen at 55% *CXCL12* methylation, comparable with the results shown for the test case control series. Aiming at reproducing the characteristics of pathological m*CXCL12* distribution in PCa, methylation values were trichotomized to obtain qualitative results. Two optimized cut-offs were introduced at 33.06% and 70.68%; m*CXCL12*_low_, m*CXCL12*_medium_, and m*CXCL12*_high_ referring to the respective groups.

Immunohistochemical staining revealed a strong nuclear and cytoplasmic positivity of basal cells in normal prostate parenchyma. In carcinomatous glands, in contrast, CXCL12 expression was altogether fainter (Supplementary Figure 1). A significant association of CXCL12 staining intensity with categorical m*CXCL12* was found (r = −0.21; *p* = 0.047; Χ^2^ = 3.97; *p* = 0.046).

### *CXCL12* methylation and biochemical recurrence-free survival analyses: Training cohort

**Figure 3:**
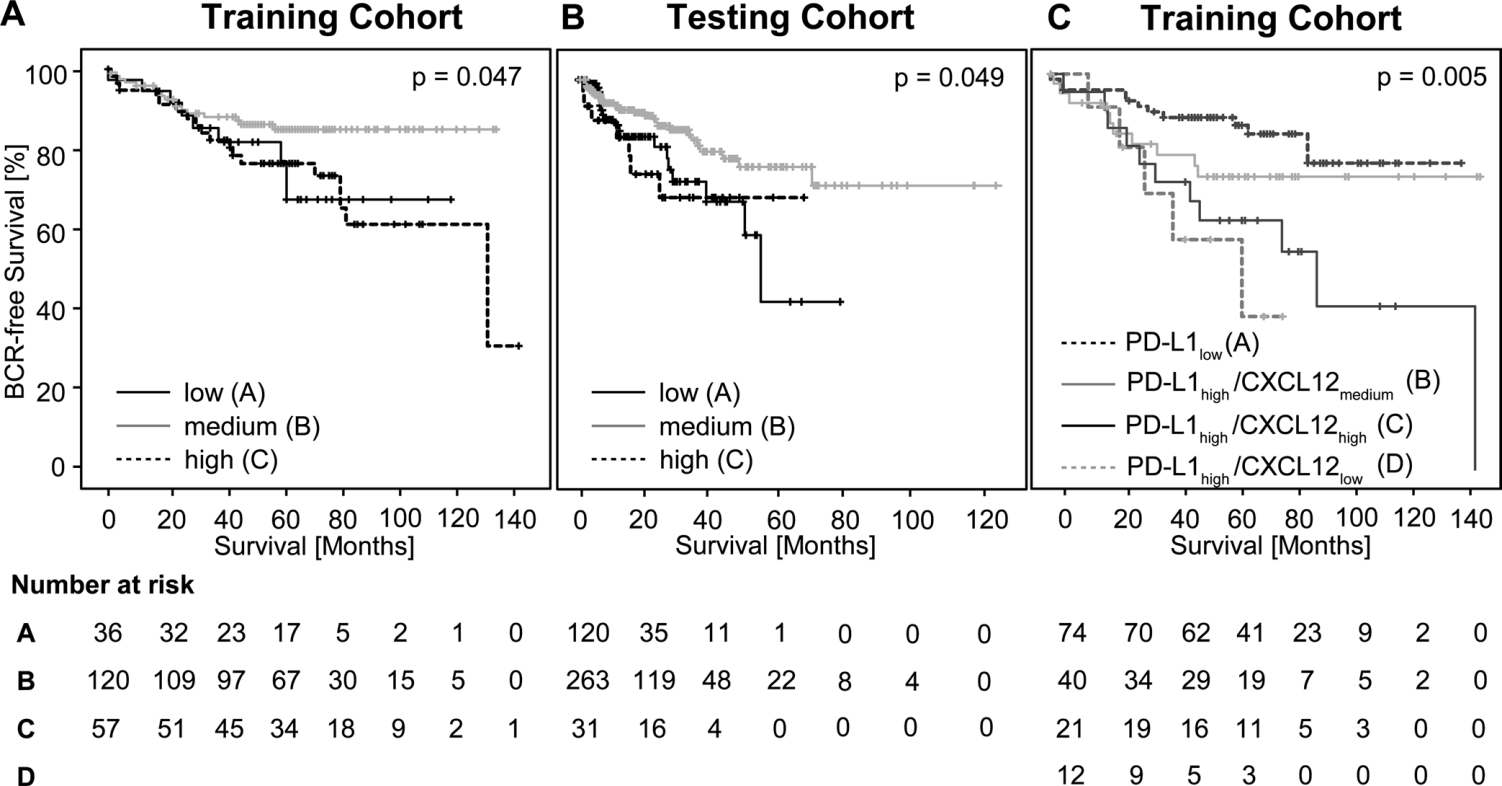
Kaplan-Meier survival of BCR-free survival in prostate cancer patients treated with radical prostatectomy and stratified by trichotomized *CXCL12* DNA methylation levels (m*CXCL12*_low_, m*CXCL12*_medium_, and m*CXCL12*_high_) in the training cohort (A) and in the testing cohort (B) Kaplan-Meier survival analysis of BCR-free survival in prostate cancer patients stratified according to the *CXCL12* methylation and PD-L1 expression levels in the training cohort (**C**), respectively.

Subsequently, we analyzed whether m*CXCL12* allows for the stratification of patients at risk for biochemical recurrence (BCR, Table 2). Trichotomized m*CXCL1*2 stratified patients according to BCR in the Kaplan-Meier analysis (χ^2^ = 6.13, *p* = 0.047, Figure 3A).

**Table 2:** Univariate and multivariate Cox proportional hazards analyses on BCR-free survival of the training and the testing cohort comprised of prostate cancer patients treated by radical prostatectomy

	Training Cohort				Testing Cohort			
	Univariate Cox		Multivariate Cox		Univariate Cox		Multivariate Cox	
Clinico-pathologic parameter / biomarker	Hazard ratio [95% CI]	*p*-value	Hazard ratio [95% CI]	*p*-value	Hazard ratio [95% CI]	*p*-value	Hazard ratio [95% CI]	*p*-value
Tumor stage (pT3 and pT4 vs. pT2)	2.84 [1.57–5.14]	0.001			5.37 [2.14–13.5]	0.001		
Tumor stage (pT3b and pT4/R1 vs. pT3a and pT2/R0 )	3.47 [1.74–6.91]	< 0.001	2.21 [1.04–4.72]	0,041	1.99 [1.13–3.49]	0.016	1.10 [0.57–2.12]	0.77
Surgical margin (R1 vs. R0)	2.46 [1.35–4.51]	0.003			1.49 [0.87–2.56]	0.15		
Gleason grading group	1.90 [1.54–2.34]	< 0.001	1.73 [1.38–2.18]	< 0.001	1.69 [1.34–2.13]	< 0.001	1.62 [1.22–2.14]	0.001
Nodal status (pN1 vs. pN0)	2.18 [0.86–5.55]	0.102			1.84 [1.00–3.35]	0.049	1.24 [0.65–2.36]	0.54
Preoperative PSA level < 4 ng/ml vs. 4–10 ng/ml, vs. > 10 ng/ml	1.70 [0.99–2.92]	0.053			1.54 [0.98–2.43]	0.060		
*CXCL12* methylation (m*CXCL12*_low/high_vs. m*CXCL12*_medium_)	2.11 [1.15–3.87]	0.016	1.48 [0.79–2.77]	0,22	1.92 [1.12–3.27]	0.017	1.76 [1.00–3.09]	0.049

For univariate Cox proportional hazard analysis, m*CXCL12*_low_ and m*CXCL12*_high_ were combined in one group. In the training cohort, 44.5% of patients fell within the m*CXCL12*_low/high_ group and 55.5% fell within the m*CXCL12*_medium_ group. Abnormal m*CXCL12*_low/high_ was associated with significantly shorter BCR-free survival (hazard ratio (HR) = 2.11 [95%CI: 1.15–3.87],*p* = 0.016, Table 2).

### Clinical performance of m*CXCL12* in the testing cohort

Table 1 displays the full clinicopathological characteristics of the testing cohort. The results from the testing cohort are entirely based upon data generated by the TCGA Research Network: http://cancergenome.nih.gov/. m*CXCL12* significantly correlated with age (*p* = 0.004), Gleason grading group (*p* = 0.034), and surgical margins/pT3b-pT4 categories (*p* = 0.005).

According to the approach chosen for the training cohort, m*CXCL12* was trichotomized using optimized cut-offs (Table 1). Trichotomized m*CXCL1*2 stratified patients according to BCR in the Kaplan-Meier analysis (χ^2^ = 6.01, *p* = 0.049, Figure 3B).

Analogous to the approach in the training cohort, m*CXCL12*_low_ *and mCXCL12* high groups were combined to m*CXCL12*_low/high_ for univariate Cox proportional hazard analysis. In the testing cohort, 39.6% of patients fell within the m*CXCL12*_low/high_ group and 60.4% fell within the m*CXCL12*_medium_ group.

**Figure 4:**
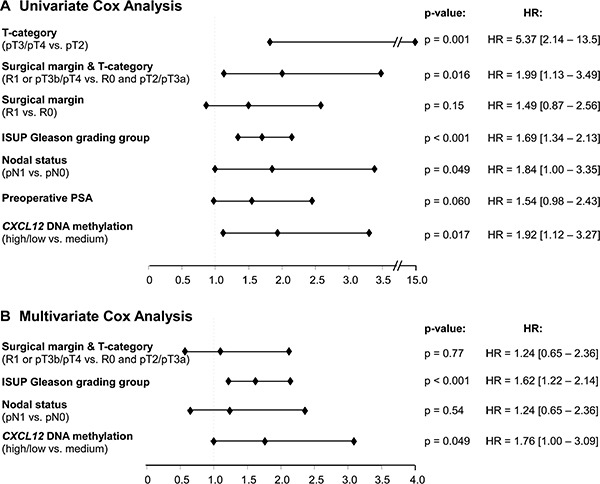
Forest Plot of the univariate (A) and multivariate (B) Cox proportional hazards analysis of BCR-free survival in the testing cohort

In univariate Cox proportional hazard analysis, m*CXCL12*_low/high_ was associated with significantly shorter BCR-free survival (HR = 1.92 [95%CI: 1.12–3.27], *p* = 0.017, Table 2, Figure 4A). In multivariate analysis including pT/R, pN, and Gleason grading group, m*CXCL12*_low/high_ qualified as an independent prognostic parameter (HR = 1.76 [95%CI: 1.00–3.09], *p* = 0.049, Table 2, Figure 4B).

### m*CXCL12* in relation to preoperative serum PSA

It has been shown that CXCL12 can help to distinguish between benign lesions and PCa among patients presenting with low serum PSA [20], which prompted us to evaluate m*CXCL12* with respect to the initial serum PSA in the full cohort of patients. Patients were stratified according to initial PSA levels. Mean m*CXCL12* did not differ between low-level PSA secreting (PSA < 4 ng/ml), medium-level PSA secreting (PSA ≥ 4 ng/ml, ≤ 10 ng/ml), and high-level PSA secreting (PSA > 10 ng/ml) tumors in both cohorts under investigation (Table 1). m*CXCL12*_low/high_ was significantly associated with BCR in medium-level PSA secreting PCa in the univariate Cox proportional hazard model (Table 3).

**Table 3:** Univariate Cox proportional hazards analyses on BCR-free survival of the training and the testing cohort comprised of prostate cancer patients treated by radical prostatectomy

	Training Cohort				Testing Cohort		
	Univariate Cox				Univariate Cox		
Stratification according to initial PSA	Hazard ratio [95% CI]	*n*	*p*-value	Clinico-pathologic parameter / biomarker	Hazard ratio [95% CI]	*n*	*p-*value
PSA < 4 ng/ml	ND	23		PSA < 4 ng/ml	3.46 [0.22–55.78]	53	0.38
PSA 4–10 ng/ml	2.02 [0.91 – 4.44]	150	0.082	PSA 4–10 ng/ml	2.09 [1.00–4.36]	288	0.049
PSA > 10 ng/ml	ND	74		PSA > 10 ng/ml	1.55 [0.67–3.59]	154	0.3

Patients were stratified according to *CXCL12* DNA methylation and BCR-free survival was analyzed in three subgroups of patients: PSA < 4 ng/ml, PSA 4–10 ng/ml, and PSA > 10 ng/ml.

ND: not determined due to low number of events.

### Association with PD-L1 expression in the training cohort

Very recently, Gevensleben *et al.* showed that PD-L1 expression served as an independent prognostic marker in PCa in the same cohort of patients [21]. We correlated m*CXCL12* with the previously published PD-L1 expression data. Mean m*CXCL12* did not differ between PD-L1_high_ and PD-L1_low_ PCa. However, Spearman's rank correlation revealed a trend towards an association of PD-L1with m*CXCL12* (ρ = 0.132, *p* = 0.084). Therefore, subgroups were defined according to PD-L1 expression in PCa: PD-L1_high_ PCa were flagged and allocated to m*CXCL12*_low_, m*CXCL12*_medium_, and m*CXCL12*_high_ groups, respectively. A total of 85 patients presented with PD-L1_low_, while 87 PD-L1_high_ patients were split into 15 m*CXCL12*_low_, 45 m*CXCL12*_medium_, and 27 m*CXCL12*_high_. In Kaplan-Meier analysis, PD-L1_low_ PCa showed the longest BCR-free survival (mean estimated 112 months), while m*CXCL12*_medium_ showed best BRC-free survival rates among PD-L1_high_ PCa (mean estimated 107 months), and PD-L1_high_/m*CXCL12*_low_, and PD-L1_high_/m*CXCL12*_high_ showed short BCR-free survival times (mean estimated 52 months and 83 months, respectively; n = 151, χ^2^ = 12.99; *p* = 0.005; Figure 3C).

### Concordance, accuracy, and robustness of m*CXCL12* detection in biopsies and ectomies

**Figure 5:**
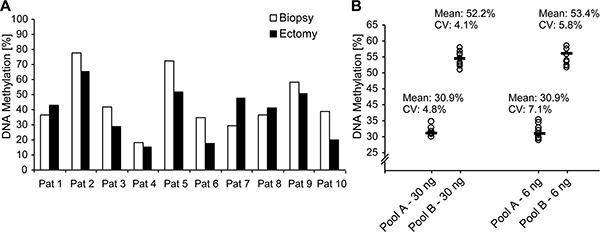
*CXCL12* DNA methylation on matching biopsies and ectomy samples from ten PCa patients (**A**) Performance evaluation of the *CXCL12* DNA methylation assay with two DNA pools. (**B**) Pool A was comprised of the five low methylated ectomy samples and pool B of the five highly methylated ectomy samples. Ten replicates were measured for each pool with two different input amounts (6 and 30 ng per reaction).

It has previously been shown that methylation assays precisely report methylation status even in formalin-fixed paraffin-embedded (FFPE) tissues [22, 23]. In order to test the utility of our assay for the analysis of biopsy specimens, we evaluated the robustness, accuracy, and concordance of m*CXCL12* testing in small biopsy specimens and the matched ectomy in a biospy test case series including 10 patients. Therefore, we correlated m*CXCL12* obtained from pooled biopsy cores of an individual patient with m*CXCL12* detected in his total tumor volume after radical prostatectomy (Figure 5A). m*CXCL12* levels detected in biopsies significantly correlated with those obtained from the total tumor volume (r = 0.76; *p* = 0.019) indicating that m*CXCL12* in biopsies represents the methylation level found in the tumor. In a second step, we tested whether m*CXCL12* can robustly and accurately be tested in small samples and over a wide range of DNA yield. Patients of the biopsy test case series were divided into two groups: five patients with m*CXCL12* ranging from 15.5% – 41.2% were assigned to group A and 5 patients with m*CXCL12* ranging from 42.7%–65.2% were allocated to group B. Tumor DNA samples of each group were pooled and measured tenfold with two different input amounts (30 ng and 6 ng, respectively) as displayed in Figure 5B. Mean m*CXCL12* was independent from the amount of DNA applied (T = 0.23, *p* = 0.82 for pool A and T = 0.62, *p* = 0.55 for pool B). The coefficients of variance of paired results neither differed in pool A nor in pool B (Levene test of homoscedastics: F = 1.76, *p* = 0.20 for pool A and F = 0.25, *p* = 0.62 for pool B, respectively).

## DISCUSSION

Recent studies have attracted notice to the CXCR4/CXCL12 axis in metastasized PCa [24, 25]. In breast and colon cancer, lower intrinsic levels of the chemoattractant chemokine CXCL12 due to hypermethylation of its promoter disrupt cellular feedback mechanisms to internalize membranous CXCR4 [14, 15]. This, in consequence, results in higher tumor cell motility and an augmented capability for metastasis [14, 15]. Our data support the postulate, that *CXCL12* methylation down-regulates tumor intrinsic CXCL12 protein expression in PCa as well, thereby fostering metastasis.

Due to its negative effect on CXCR4 expression, tumor intrinsic CXCL12 may be a valuable biomarker in clinical situations that require decision-making. Immunohistochemical CXCL12 staining, however, did not discriminate between PCa with favorable and adverse outcome in the present study. Abnormal methylation of the *CXCL12* gene, in contrast, was associated with a shorter BCR-free survival of primary PCa. In two independent cohorts of patients after radical prostatectomy, *CXCL12* hypo- and hypermethylation served as a prognostic biomarker identifying patients with early BCR and qualified as an independent prognostic factor in the testing cohort.

The fact that the cohort is based on radical prostatectomy specimens, however, is a major limitation of this study. Gene methylation can be robustly determined even in little formalin-fixed pathological material [22, 23]. If the prognostic value of *CXCL12* methylation can be transferred to a biopsy-based cohort, its analysis may be a promising tool in order to identify patients with innocuous tumors that can safely be followed by active surveillance protocols. Conversely, aberrant methylation of *CXCL12* may indicate a high metastatic potential. In consequence, affected patients possibly will benefit from radical treatment, including radical prostatectomy. In a paired analysis of *CXCL12* methylation based on biopsy and matched ectomy specimens, we were able to show that the results of m*CXCL12* assessments are concordant. In addition, our assay seems to be able to reliably determine *CXCL12* methylation in biopsy material. The variation coefficients of our results were slightly higher the lower the amount of applied DNA was, however, in homoscedastic testing, no difference could be observed. In practice, growing variances in small samples may easily be counteracted by increasing the number of replicates in the analysis. If the assay will be established for future routine use, a broad spectrum of biopsy material will be needed to precisely set the measuring range and achieve tight tolerances.

Immunotherapy has been emerging as a promising treatment strategy in cancer patients [26–28]. Among major immune checkpoints, e.g. PD-1 and CTLA4, the pharmacologic blockage of CXCR4 and its endogenous ligand CXCL12 has appeared as a prime target for blocking strategies in the treatment of cancer patients [10]. Recent studies have focused on pharmacological CXCR4 inhibition within the cross relation of anti-PD-1/anti-PD-L1 drug resistance [29, 30]. *In vitro* and murine *in vivo* studies have provided data that ADM3100 induced blockage of CXCR4 restores sensitivity to PD-1 and CTLA-4 checkpoint inhibitors in anti-PD1 and anti-CTLA-4 drug-resistant tumors and thus might help to overcome resistance to immune therapy [10, 18, 30]. Consequently, a combined anti-CXCR4/CXCL12 and anti-PD1/PD-L1 (Ulocuplumab, BMS-936564/Nivolumab, BMS-936558) therapy is under clinical investigation in metastatic solid tumors (CXCessoR4 trial, ClinicalTrials.gov Identifier: NCT02472977). In our study, patients with high PD-L1 expression and aberrant *CXCL12* methylation presented with significantly shorter BRC-free survival intervals than patients with either low PD-L1 expression or high PD-L1 expression plus normal *CXCL12* methylation. Accordingly, a combined anti-PD-L1/PD and antagonistic CXCR4/CXCL12 treatment seems to be a promising approach in PCa as well. For the identification of eligible patients, methylation of *CXCL12* together with immunohistochemical staining of PD-L1 might be a powerful tool. Whether carcinomatous *CXCL12* methylation predicts responsiveness to therapeutic intervention targeting the CXCR4/CXCL12 axis remains the object of future research.

## MATERIALS AND METHODS

### Subjects

### Patients and clinical endpoint

1. The test case control series included 66 FFPE tissue samples from prostate tissue of 25 PCa patients. The samples included 25 prostate cancers, 24 normal adjacent tissue (NAT) and 22 benign prostatic hyperplasia (BPH) specimens.

2. Patient training cohort: A patient cohort comprised of 247 patients with histologically confirmed adenocarcinoma of the prostate who underwent radical prostatectomy at the University Hospital Bonn between 1998 and 2008 was retrospectively enrolled. BCR-free survival was considered as the primary endpoint of the study.

3. Patient testing cohort: The TCGA cohort provided data of 498 patients with histologically confirmed adenocarcinoma of the prostate. Furthermore, transcription data were available from additional 50 specimens obtained from patients with simultaneous PCa. BCR-free survival was considered as the primary endpoint of the study.

4. The biospy test case series included 10 patients with primary PCa diagnosed at the University Hospital Bonn based on 4 to 12 transrectal core needle biopsies. All patients underwent radical prostatectomy during the course of their disease.

### Choice of illumina human methylation450 bead

*CXCL12* methylation was available from 498 PCa specimens and 50 normal tissues. Clinical follow-up was provided in 417 individuals (mean follow-up period 22 months, range 1–115 months). The TCGA methylation data has been created by TCGA Research Network: http://cancergenome.nih.gov/ using the Infinium HumanMethylation450 BeadChip (Illumina, Inc., San Diego, CA, USA). Methylation values for each bead pair comprised of a variant specific for the methylated and the unmethylated status, respectively, were calculated using the formula 100*Intensity_Bead_Methylated/(Intensity_Bead_Methylated + Intensity_Bead_Unmethylated). Bead pair cg26267854 located within the upstream CpG-island of the *CXCL12* promoter was selected.

### Ethics

The Institutional Review Board (IRB) at the University Hospital of Bonn approved the study (Lfd. Nr. 071/14). Informed consent has been obtained from all patients that were included in the TCGA cohort in accordance with the Helsinki Declaration of 1975.

### Sample preparation

For methylation analysis, ectomy samples were processed according to the InnuConvert Bisulfite All-In-One Kit (Analytik Jena, Germany) as previously published [31]. Bisulfite DNA from biopsies were prepared as described earlier [23]. Length of tumor infiltrate in one tissue biopsy ranged from 0.1 mm to 12 mm (median 4 mm). Number of tumorous cores in one set of biopsies ranged from 1–8 (median 4). Tumorous tissue was marked, micro-dissected, and further processed as described above. For comparison with methylation results obtained from matched biopsies, total tumorous tissue from radical prostatectomy specimens was micro-dissected and bisulfite converted.

For the analytical performance verification of the assay, a dilution series of bisulfite-converted artificially methylated DNA (CpGenome™ Universal Methylated DNA; Merck Millipore, Darmstadt, Germany) and unmethylated DNA (NW Andrology & Cryobank Inc., Spokane, WA, USA) was used.

### *CXCL12* quantitative methylation-specific real-time PCR

Gene methylation of *CXCL12* was quantified by a quantitative-methylation real-time PCR assay, with the primers and probes shown in Table 4. The assay was performed using an AB 7500 Fast Real-Time PCR System (Life Technologies Corporation, Carlsbad, CA, USA). If not stated otherwise in the result section, DNA input was 25 ng of bisulfite-converted DNA from FFPE ectomy tissue for each PCR reaction. 5 μl DNA from biopsy samples was applied without prior quantification. A calibrator sample (50:50 mixture of bisulfite-converted artificially methylated and unmethylated DNA) was used. Each sample was measured in triplicate. Sample measurements were considered valid when the following quality criterion was met:

**Table 4:** Primer and probe sequences of the quantitative *CXCL12* methylation-specific real-time PCR

Primer/Probe name	Primer/Probe sequence
*CXCL12*-F	5′- attggagagattgaggatttta -3′
*CXCL12*-R	5′- aaactaacttatttacttttcatta -3′
*CXCL12*-P-M	5′- Atto647N-cgccatcgaaaaaccccgtccc-BHQ-2 -3′
*CXCL12*-P-U	5′- HEX-caccatcaaaaaaccccatccc-BHQ-1 -3′

CT_*CXCL12*-P-U_ or CT_*CXC12*-P-M_ < 35

Other samples were excluded from the analysis due to insufficient DNA yield. The *CXCL12* methylation was calculated using the ΔΔCT method:

ΔCT = Δ_*CTCXCL12*-P-U_ – ΔCT _*CXCL12*-P-M_, ΔΔCT = ΔCT_sample_ – ΔCT_calibrator_, m*CXCL12* = 100%/(1 + 2^ΔΔCT^).

### Immunohistochemical analyses of PD-L1 expression and *CXCL12* expression

PD-L1 expression data were collected from our previous study [21]. In brief, the anti-PD-L1 antibody clone EPR1161(2) (Abcam, Cambridge, UK) was used. Specific membranous and cytoplasmic staining of epithelial tumor cells was considered positive [21]. The intensity of PD-L1–staining was scored semi-quantitatively as negative (0), weak (1), moderate (2), or strong (3) by two independent and blinded pathologists. For each patient a PD-L1 expression value was computed. Up to five cores per patient were scored semiquantitatively and mean averaged. Patients’ stratification for Kaplan-Meier analysis was conducted using the median PD-L1 expression of the total cohort.

For immunohistochemical CXCL12 staining, TMA blocks were cut (3 μm) and mounted on Superfrost slides (Menzel Gläser, Braunschweig, Germany). A polyclonal anti-CXCL12 antibody (bios #bs-4938R, 1:600) was used. A Medac Autostainer 480 performed the staining (Medac, Wedel, Germany). Antigen-antibody-binding was visualized by horse-radish-peroxidase polymer method. To assess the specificity of staining, negative controls with PBS were run.

Ninety-one cases could be evaluated in immunohistochemical CXCL12 staining. CXCL12 staining was observed in the nuclei as well as in the cytoplasma of basal cells of PCa glands. Nuclear and cytoplasmic staining was assessed as negative (0), weak (1), or strong (2). Scores were added and averaged over 2–3 tissue scores. For correlation analysis and survival analysis, scores were dichotomized by the median of the total cohort.

### Statistical analyses

Statistical analysis was performed using SPSS, version 22 (SPSS Inc., Chicago, IL). The relationship between input DNA methylation and measured DNA methylation was assessed by linear regression. In the training and testing cohort, comparative studies of dichotomized *CXCL12* methylation values were tested using the Mann-Whitney *U* test for paired analysis and using the Kruskal-Wallis test in cases when more than two groups were compared. Statements regarding potential correlations were made using the Spearman's correlation coefficient ranked between two assessed variables. Comparative analysis of methylation in the biopsy case test series were performed using the Pearson correlation coefficient. Diluted tumor DNA of the biopsy case test series were analysed using the *t*-test and the Levene test of homoscedastics. BCR-free survival estimates were calculated according to Kaplan-Meier and Cox proportional hazards regression analysis. Multivariate analysis was performed with all parameters tested significant in the univariate analysis. *P*-values (two-sided) lower than 0.05 were considered significant. The test case control series was analysed using GraphPadPrism (GraphPad, La Jolla, CA). For comparison of independent groups of the test case control study, one-way analysis of variance (one-way ANOVA including Barlett’ statistic) with Bonferroni-post-hoc testing was applied.

## SUPPLEMENTARY MATERIAL FIGURE


