# Supplementary material for: CXCL12 promoter methylation and PD-L1 expression as prognostic biomarkers in prostate cancer patients
Source: Oncotarget. 2016 Jul 22;7(33):53309–20. doi: 10.18632/oncotarget.10786 (PMC5288188; doi:10.18632/oncotarget.10786)
Supplement: Supplementary file 1 [file oncotarget-07-53309-s001.pdf]

CXCL12 promoter methylation and PD-L1 expression as prognostic biomarkers in prostate cancer patients

Supplementary Materials

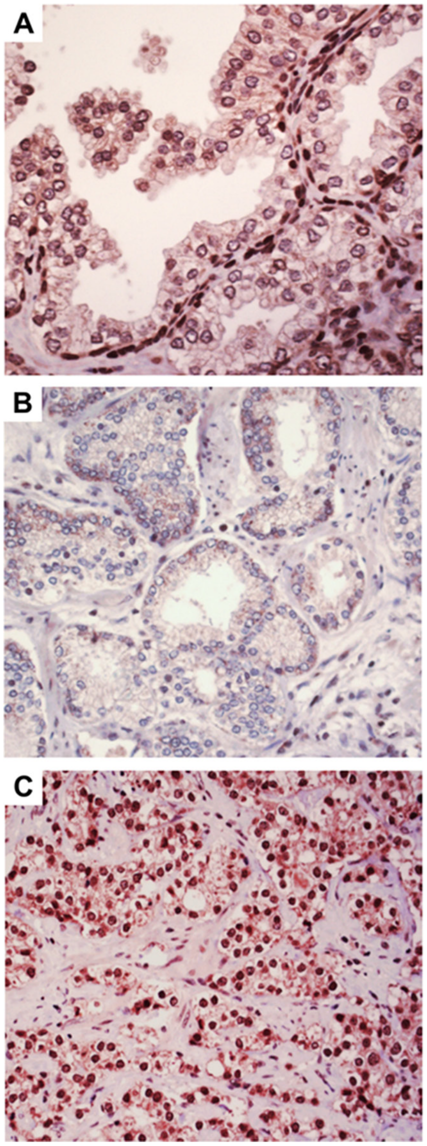

Supplementary Figure S1: Immunhistochemical staining of CXCL12 in prostate cancer from the training cohort. Representative stainings are shown of normal tissue (**A**), and PCa with negative staining (**B**) and positive staining (**C**).
